# A Comprehensive Review of Bioinformatics Tools for Genomic Biomarker Discovery Driving Precision Oncology

**DOI:** 10.3390/genes15081036

**Published:** 2024-08-06

**Authors:** Alexis J. Clark, James W. Lillard

**Affiliations:** Department of Microbiology, Biochemistry, and Immunology, Morehouse School of Medicine, Atlanta, GA 30310, USA; ajclark@msm.edu

**Keywords:** oncology, bioinformatics, biomarker discovery, predictive algorithms, RNA-Seq

## Abstract

The rapid advancement of high-throughput technologies, particularly next-generation sequencing (NGS), has revolutionized cancer research by enabling the investigation of genetic variations such as SNPs, copy number variations, gene expression, and protein levels. These technologies have elevated the significance of precision oncology, creating a demand for biomarker identification and validation. This review explores the complex interplay of oncology, cancer biology, and bioinformatics tools, highlighting the challenges in statistical learning, experimental validation, data processing, and quality control that underpin this transformative field. This review outlines the methodologies and applications of bioinformatics tools in cancer genomics research, encompassing tools for data structuring, pathway analysis, network analysis, tools for analyzing biomarker signatures, somatic variant interpretation, genomic data analysis, and visualization tools. Open-source tools and repositories like The Cancer Genome Atlas (TCGA), Genomic Data Commons (GDC), cBioPortal, UCSC Genome Browser, Array Express, and Gene Expression Omnibus (GEO) have emerged to streamline cancer omics data analysis. Bioinformatics has significantly impacted cancer research, uncovering novel biomarkers, driver mutations, oncogenic pathways, and therapeutic targets. Integrating multi-omics data, network analysis, and advanced ML will be pivotal in future biomarker discovery and patient prognosis prediction.

## 1. Introduction

In recent years, high-throughput technology has become increasingly available due to advancements in next-generation sequencing (NGS) to analyze single nucleotide polymorphisms (SNPs), copy number variations, gene expression, microRNA expression, protein expression, as well as other genetic alterations. Precision oncology utilizes high-throughput technologies and bioinformatics tools to tailor cancer treatments to individual patients on the basis of their unique genetic profiles. This approach improves the ability to identify and validate biomarkers significant to cancer diagnosis, predicting outcomes, and developing personalized therapeutic plans. Integrating bioinformatics in precision oncology requires expertise in the fields of oncology, bioinformatics, and biostatistics.

For example, genomic profiling with NGS technologies such as whole-genome sequencing (WGS) and whole-exome sequencing (WES), allows for rapid and comprehensive analysis of genetic mutations, SNPs, and structural variations within tumors. Bioinformatics tools play a critical role in integrating and interpreting data from these technologies. For instance, tools for variant annotation and interpretation, such as ANNOVAR, help predict the functional consequences of genetic variants, aiding in the identification of actionable mutations. Additionally, pathway analysis tools like Ingenuity Pathway Analysis (IPA) and Gene Set Enrichment Analysis (GSEA) identify affected biological pathways and networks, offering novel insights into tumorigenesis.

The process of biomarker discovery and validation is fundamental in precision oncology. Bioinformatics facilitates the identification of biomarkers that predict treatment response or disease progression, such as the immunotherapy target Programmed Death Ligand 1 (*PD-L1*) [1]. Other examples of biomarkers used in current therapies include epidermal growth factor receptor (*EGFR*) inhibitors for non-small cell lung cancer patients with *EGFR* mutations and PARP inhibitors in cancers with Breast Cancer Gene 1/2 (*BRCA1/2*) mutations. These biomarkers undergo stringent validation processes to ensure their reliability and clinical utility.

As researchers improve biomarker identification methods and expand large data repositories, the complexity and volume of the data require more powerful analytical tools. This has led to the increasing demand for machine learning (ML) and predictive algorithms. ML algorithms are adept at managing and interpreting large, high-dimensional datasets and identifying patterns and relationships traditional methods might overlook. Techniques such as dimensionality reduction and feature selection allow ML algorithms to efficiently process and analyze biological data, evaluate disease mechanisms, and identify potential biomarkers.

Despite the promise of these advanced tools, several challenges remain, including data acquisition, quality control, and accurate reporting. Applying bioinformatics in diagnosis, preventative medicine, and personalized treatment plans requires a multidisciplinary approach. The use of bioinformatics in precision oncology is transforming cancer treatment, facilitating the discovery and application of biomarkers amidst the challenges posed by complex data.

## 2. Overview of DNA Sequencing Methods

DNA sequencing is utilized to enhance medical research. Key genomic research events, such as chain termination (Sanger sequencing) in 1977 and polymerase chain reaction (PCR), underlined significant studies such as the 1990–2003 Human Genome Project [2,3]. Over time, new cost-effective methods for WGS and WES have emerged [4]. 

### 2.1. Whole Genome Sequencing

The human genome is comprised of approximately 3 billion base pairs, including both coding and non-coding regions [5]. When exploring the genome, it is important to consider distinctions between introns and exons. Introns are the non-coding regions of genes, while exons are the coding regions that are responsible for the proteins produced in an organism.

WGS is a comprehensive analysis technique that involves sequencing the entire DNA content of an organism, enabling the identification of all genetic variants and providing insights into the complete genomic composition. This method unveils genetic variants such as SNPs and larger structural variations that contribute to the genetic makeup of an organism. SNPs are the most common type of genetic variation among people. They are representative of a difference in a single nucleotide. These variations can occur in both coding and non-coding regions of the genome and can influence how humans develop diseases [6,7].

**Figure 1:**
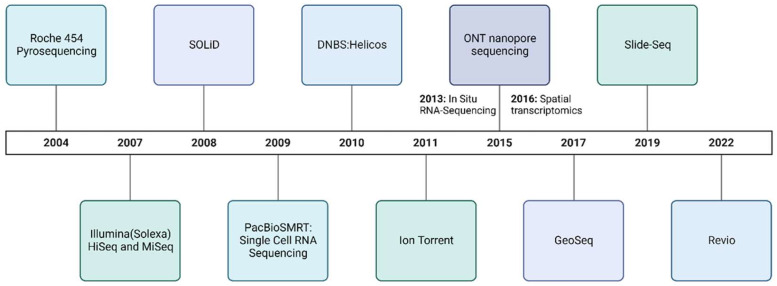
**Overview of sequencing technologies 2004–2022.** Post-Sanger sequencing technologies, beginning with Roche 454 Pyrosequencing in 2004, Illumina HiSeq and MiSeq (2007), SOLiD (2008), PacBioSMRT (2009), DNBS: Helicos (2010), Ion Torrent (2011), in situ RNA sequencing (2013), ONT nanopore (2015), spatial transcriptomics (2016), GeoSeq (2017), Slide-Seq (2019), and Revio (2022), illustrate the continuous improvements contributing to the genomics. It explores the various NGS sequencing techniques that have expanded upon chain termination and polymerase chain reaction.

DNA sequencing is important in bioinformatics biomarker discovery studies because it allows for the comprehensive analysis of genomic information. By examining DNA sequences produced by methods like those mentioned in Figure 1, researchers identify genetic variations, mutations, and specific markers associated with various disease phenotypes. This information is crucial in understanding the genetic basis of diseases, predicting predispositions, and discovering biomarkers that can serve as indicators or responses to treatments. In addition, it provides insights into the relationships between genetic variations and diseases, enabling the identification of potential biomarkers for diagnostics, prognostics, and personalized medicine.

### 2.2. Whole-Exome Sequencing (WES) and RNA Sequencing (RNA-seq)

While WGS involves sequencing all DNA content, including coding and non-coding regions, WES focuses on selectively sequencing the protein-coding regions of known genes [4]. WES is an NGS method that utilizes sequencing to comprehensively investigate protein-coding regions of the genome along with ~20 nt of connecting introns [4]. WES covers over 95% of the exons in the genome, including 85% of disease-causing mutations and predisposing SNPs [8,9,10].

Next-generation sequencing (NGS) has evolved rapidly in recent years due to increased throughput and reduced cost compared to traditional methods like Sanger sequencing [11,12,13]. Figure 1 highlights some of the NGS platforms developed in recent years. The evolution of NGS technologies in three distinct generations emphasizes the significant advancements in sequencing efficiency, accuracy, and throughput. First-generation sequencing technologies are based on Sanger sequencing techniques. Sanger sequencing was developed by Frederick Sanger in the 1970s [14]. This method uses chain-terminating dideoxynucleotides during DNA synthesis, resulting in fragments of varying lengths that are then separated by electrophoresis [14]. While revolutionary, this method is limited by its low-throughput, high-cost, and labor-intensive process and can only sequence one DNA fragment at a time. Since then, sequencing technologies have evolved to improve accuracy and reproducibility in its experiments.

Second-generation NGS is based upon pH detection or pyrosequencing and may be referred to as first-generation massively parallel sequencing (MPS). These methods are used by technologies such as Illumina, 454 pyrosequencing, and Ion Torrent. These technologies involve sequencing by synthesis (SBS) where the addition of nucleotides is detected in real-time, either by light emission or pH changes or sequencing by hybridization [15,16]. For example, Roche/454 FLX Pyrosequencer was the first high-throughput screening platform used in 2004 [17,18]. In pyrosequencing, as nucleotides are incorporated by DNA polymerase, a pyrophosphate cascade is initiated that produces light by luciferase [16]. The resulting light is proportional to the quantity of nucleotides added [16]. Sequencing by hybridization utilizes overlapping oligonucleotide sequences to determine the DNA sequence. In SBS, a primer attaches to the adapter binding site of the forward strands, and a polymerase adds fluorescently tagged dNTP to the DNA strand. This led to the development of ion and Illumina Genome Sequencing, which utilizes the principles of SBS while improving technology sensitivity [19]. SOLiD is another sequencing technology that uses emulsion PCR with small magnetic beads to amplify fragments and DNA ligase for sequencing the fragments [16,19,20]. The major advancements brought by second-generation sequencing are increased throughput, reduced cost per base, and the ability to sequence millions of DNA fragments simultaneously. However, these technologies require an amplification step before sequencing. This ensures that there is a sufficient quantity of DNA template for base additions. Some common amplification techniques are emulsion PCR, bridge amplification, and DNA nanoball generation. This can introduce biases and errors. Additionally, they generate shorter reads compared to Sanger sequencing, which can make complex genome assembly more difficult. Second-generation NGS is most commonly used, and these technologies require an amplification step before sequencing. 

Lastly, third-generation sequencing is referred to as second-generation MPS and can include Single-Molecule Real-Time (SMRT) sequencing and nanopore sequencing methods like the technologies developed by PacBio and Oxford Nanopore. These technologies read single DNA molecules in real time without the need for amplification. PacBio SMRT is a long-read sequencing technology developed by Pacific Biosciences. It uses circular consensus sequencing (CCS) to generate highly accurate read lengths between 10–15 kb. The long reads are beneficial for investigating genomic regions, such as repetitive sequences and structural variants. Similarly, Oxford Nanopore Technologies developed nanopore sequencing that encompasses distinct features, including long-reads exceeding 100 kb, real-time sequencing, portable devices like MinION, and is useful in a wide range of applications, like assembling complete genomes. Oxford Nanopore improves accuracy through algorithmic advancements and base-calling software. The significant improvements of third-generation sequencing are the elimination of amplification bias, the ability to generate longer reads, and real-time data acquisition. This allows for immediate analysis and faster turnaround times. Despite these advancements, third-generation sequencing initially faces high error rates compared with second-generation sequencing methods. Additionally, while costs are decreasing, third-generation sequencing is still relatively expensive compared with second-generation methods. Although these technologies made great strides in genomic research, there is still room for improvement and implementation in bioinformatic pipelines.

RNA-seq is a technique that uses NGS to examine the quantity and sequences of RNA in a sample, enabling the study of gene expression, transcriptome structure, and regulation. It allows for the detection of known and novel features in a single assay, such as transcript isoforms, gene fusions, and single nucleotide variants, without the limitation of prior knowledge. The RNA-seq workflow typically includes steps such as RNA extraction, reverse transcription into cDNA, adapted ligation, amplification, and sequencing. This approach evolved alongside technological advancements (i.e., NGS), leading to the development of diverse computational tools and methodologies that help unlock the full potential of RNA-seq data. Indeed, RNA-seq has become the preferred method for studying the transcriptome, offering advantages over previous technologies, and is widely used in various fields, including gene regulation studies and disease research.

## 3. Overview of RNA-Seq Bioinformatics

RNA-Seq bioinformatics workflows involve computational steps to process, analyze, and interpret RNA sequencing data. It is utilized to understand molecular mechanisms in cancer. 

### 3.1. Data Quality Control and Pre-Processing

**Figure 2:**
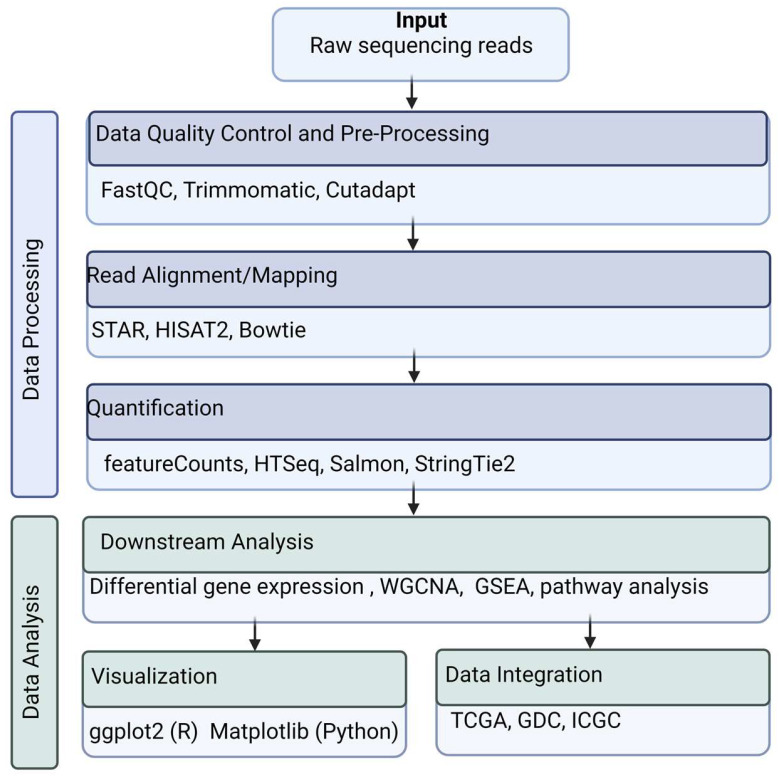
**Overview of RNA-Seq data workflow**. In this example of RNA-Seq data analysis workflow, raw sequence reads serve as the input. Raw sequences undergo pre-processing and quality control. Next, the Fastq files are aligned to a reference genome (SAM/BAM files) and are quantified to generate count matrices (Text files). Downstream analysis, such as differential gene expression, uses the count matrices as input. The data results can be visualized and integrated using R packages, Python libraries, and other tools.

Transcriptomic data undergoes several transformations before it is used in analytical pipelines (Figure 2). After data acquisition, pre-alignment quality control assesses the sequencing quality and contamination screening using tools like FastQC. FastQC analyses raw data to identify adapters and low-quality reads [21]. It conducts its quality assessment by summarizing the per-base and per-sequence quality scores, per-sequence GC content, per-sequence adapter content, per-sequence adapter content, per-sequence read lengths, and overrepresented sequences. Adapters are synthetic sequences that should be removed prior to alignment. These can be filtered and trimmed using tools such as Trimmomatic or Cutadapt [22,23]. Trimmomatic is a flexible and efficient tool used to trim paired-end data [22]. Cutadapt locates and removes adapter sequences, primers, poly-A tails, and other unwanted sequences from the reads [23].

### 3.2. Read Alignment/Mapping

Once the quality of the raw data has been assessed, genetic information cannot be interpreted in its current form. Thus, the next step is adding biological context to the raw data. This is done by aligning or mapping the reads to a reference genome. STAR, HISAT2, and Bowtie are commonly used alignment tools [24,25,26]. STAR is a high-speed alignment that aligns RNA-seq read to a reference genome. It provides accurate mapping of splice junctions and can efficiently handle large datasets [24]. HISAT2 (Hierarchical Indexing for Spliced Alignment of Transcripts) is a fast and memory-efficient aligner particularly useful for spliced alignments. It supports alignment reads to large genomes and accurately reads across splice junctions [25]. Bowtie is an extremely fast, memory-efficient aligner that is suitable for aligning short DNA sequences to a reference genome [26]. Several human reference genomes exist, such as the 2009 Genome Reference Consortium (GRC) GRCh37 and 2013 GRCh38. The transcripts can be quantified after the reads are aligned to a reference genome.

### 3.3. Quantification

Gene quantification refers to the process of measuring the expression levels of genes from high-throughput sequencing data. This process allows for analyzing specific gene activity within a biological sample. The quantification step addresses scientific questions about the identity of transcribed genes and how abundantly they are expressed in various biological conditions. It is essential for understanding gene regulation, identifying biomarkers, and studying cellular responses. RNA-Seq is the primary data source for gene expression and quantification. Microarray technology, although less commonly used today, can also be used for gene expression quantification. Several bioinformatics tools, such as featureCounts, HTSeq, Salmon, and StringTie2, can be used to generate the raw read counts [27,28,29,30]. featureCounts counts reads mapped to genomic features such as genes or exons. It is a fast and efficient tool that supports a wide range of input formats and handles both single-end and paired-end reads [27]. HTSeq also counts reads for various genomic features. It supports multiple counting modes (e.g., union, intersection) and is compatible with several alignment formats [28]. Salmon quantifies transcript abundances from RNA-seq data. It uses lightweight alignment and quasi-mapping for fast and accurate quantification. Salmon can work with both transcriptome and genome alignments and offers robust management of sequencing bias and batch effects [29]. Raw read counts are often normalized to account for variations in sequencing depth or library size. Common normalization methods include TPM (Transcripts Per Million) and FPKM (Fragments Per Kilobase of transcript per Million mapped reads). Gene quantification is a foundational step in genomics and transcriptomics research. This step serves as the basis for downstream analyses that contribute to understanding gene expression and its role in biology and disease.

### 3.4. Alternative Splicing Analysis

Alternative splicing analysis of RNA involves the detection, statistical comparison, and effect prediction of splice events, which are variations in the way exons are joined together to form a mature mRNA transcript. Various software packages and computational tools, e.g., RNA-Seq Mutational Analysis Tools (rMATS) or (Surpassing Parameter Prediction Algorithms (SUPPA) have been developed to facilitate isoform-specific alternative splicing analysis, including tools for detecting splicing alterations, testing differential splicing between two groups, and predicting the biological impact of alternative splicing [31,32]. rMATs functions by detecting alternative splicing events from either paired-end or single-end RNA-seq data. It has the ability to identify various splicing events such as exon skipping and intron retention, while providing detailed statistical analysis of splicing differences. SUPPA quantifies alternative splicing events and estimates differential splicing using transcript-level abundance to predict the splicing events. This tool can also efficiently handle large-scale transcriptomics data and provide visualization tools for splicing analysis. This analysis is crucial for understanding the diversity of gene products that can be generated from a single gene and for studying the impact of alternative splicing on gene function and regulation.

### 3.5. Visualization

During the visualization process, tools like ggplot2 (R) and Matplotlib (Python) are used to create graphs to depict patterns, differential gene expression, and splicing events. Transcriptomic data visualization involves the graphical representation of transcriptomic data to aid in the interpretation and analysis of gene expression patterns, transcript structures, and regulatory mechanisms. eVITTA is a web-based visualization and inference toolbox for transcriptome analysis that provides modules for analysis, visualization, and inference [33]. Giotto, Scanpy, Seurat, and Squidpy, are examples of advanced spatial transcriptomic technologies that provide functionalities for plotting spatial transcriptomic data in Euclidean space. They enable detailed visualizations and analysis of interactions between cells and genes in spatial transcriptomic datasets [34,35,36,37]. These tools are useful for researching the cellular interactions important for biological processes and diseases. For instance, Giotto has been successfully used to analyze imaging-based spatial transcriptomic datasets and large-scale spatial proteomic datasets. It was used to profile the spatial distribution of proteins. In pancreatic ductal adenocarcinoma (PDAC) samples, identifying distinct clusters and providing more understanding of tumor heterogeneity [38]. Phantasus is a third-party application that can expedite bulk RNA-Seq data processing and offers a graphical user interface or a programmatic interface for data visualization and analysis [39]. omicplotR is a Shiny app to visualize RNA-seq, meta-RNA-seq, and 16s rRNA data, providing methods for differential RNA expression analysis and visualization [40]. BEAVR is a browser-based tool for the exploration and visualization of RNA-seq data, which facilitates interactive analysis and exploration of RNA-seq data, allowing both novices and experts to perform differential gene expression analyses on RNA-seq datasets [41]. These tools offer diverse functionalities for visualizing and analyzing transcriptome data, catering to different research needs and user expertise levels.

### 3.6. Data Integration

RNA-Seq data may be combined with other omics data sources to comprehensively investigate biological processes and diseases. This process allows researchers to compare gene expression profiles, detect regulatory mechanisms, and gain a comprehensive understanding of the transcriptome under various biological conditions. Integration methods often involve the alignment of samples across conditions using shared highly variable genes and the comparison of cell subpopulations and their marker genes across different datasets.

## 4. Open-Source Tools 4

In recent years, efforts have been made to compile and analyze genomic data. This work led to the development of databases and publicly available tools to analyze cancer omics data for biomarker discovery. Platforms like the European Nucleotide Archive (ENA) and Sequence Read Archive (SRA) store raw data files, providing researchers access to the primary data obtained from high-throughput sequencing experiments in various formats, including Fastq and SRA. These open-source repositories are foundational for researchers offering necessary genetic information for in-depth analysis. 

Additionally, tools like Figshare and Dryad Digital Repository house a wide range of academic research data, including genomics data and cross-disciplinary datasets. Harvard Dataverse Network is a multi-disciplinary tool that stores research data and underscores collaborative efforts. Various open-source repositories such as Network Data Exchange and Open Science Framework serve as resources for scientists, enabling collaboration and exploration of data and promoting the advancement of research and data analysis. Reactome is an open-source pathway database that supports genomics analysis through integrative analytical and visualization tools. SpatialDB is a database and visualization tool for spatially resolved transcriptomes. These tools provide a range of functionalities for transcriptome analysis, including differential gene expression, sequence alignment, and annotation. 

## 5. Data Repositories

Understanding patient tumor samples is critical for identifying biomarkers and targeted therapies. The conjunction of clinical samples with clinical records enhances the characterization process. The Gene Expression Omnibus (GEO) database was established in 2001 by the National Center for Biotechnology Information (NCBI). This public repository contains over 3.3 million samples of gene expression data generated from more than 100,000 experiments (e.g., microarray, bulk RNA-seq, scRNA-seq) and from multiple organisms.

The ENCODE (Encyclopedia of DNA Elements) project was launched in September 2003 by the National Human Genome Research Institute (NHGRI). This repository provides data produced by the ENCODE Consortium, offering a comprehensive resource for the analysis of transcriptomic data. The goal of ENCODE was to create a comprehensive map of all the functional elements in the human genome, including genes, regulatory elements, and other non-coding regions.

The Cancer Genome Atlas (TCGA) is one of the largest multi-omics cancer repositories initiated in 2006 by the National Cancer Institute (NCI) and the National Human Genome Research Institute. The data comprises paired normal and tumor tissue samples from over 20,000 patients, 33 different cancer types, 7 data types, and 15 genomic assays. TCGA has generated over 2.5 petabytes of genomic, epigenomic, transcriptomic, and proteomic data. 

The Sequence Read Archive (SRA) was established in 2009 by the National Center for Biotechnology Information (NCBI), a division of the National Library of Medicine (NLM) within the National Institutes of Health (NIH). It was developed in response to the rapid growth of high-throughput sequencing data and the need for a centralized repository where researchers could deposit and access this data. This archive of high-throughput sequencing data includes DNA and RNA sequences, which provide a centralized repository for data storage and access.

The Expression Atlas was established in 2012 by the European Bioinformatics Institute (EMBL-EBI) with funding from the Wellcome Trust. This project was conceived as a response to the growing need for a centralized resource for gene expression data across various species and biological conditions. It currently holds data from over 250 million samples across 80 species, making it one of the most comprehensive repositories of gene expression data available. The platform continues to evolve and expand, incorporating new technologies and datasets to remain at the forefront of bioinformatics research.

Launched in 2012, the Registry of Research Data Repositories (re3data.org) is a global registry of research data repositories from all academic disciplines, providing an overview of existing research data repositories to help researchers identify suitable repositories for their data. Re3data covers thousands of data repositories from a wide range of disciplines, including social sciences, natural sciences, humanities, and engineering. For each repository, re3data provides detailed information, including the types of data accepted, data submission guidelines, access policies, and long-term preservation strategies. Importantly, e3data provides an API that allows developers to integrate its data into other applications.

In 2017, Genomic Data Commons (GDC) expanded the Center for Cancer Genomics (CCG) collaborative data generation model to include TCGA and Therapeutically Applicable Research to Generate Effective Therapies (TARGET) programs on its platform. The International Cancer Genome Consortium (ICGC) is a similar repository containing over 86 research projects, 22 cancer primary sites, and data from about 25,000 patients. On the ICGC portal, data can be analyzed and downloaded for multi-omics studies. Although these tools are helpful in the advancement of oncology research, the lack of clinical covariates, such as patient follow-ups and treatments, often impede the translation of genomic research to clinical outcomes. Bioinformatic tools such as the GDC portal (National Cancer Institute), cBioPortal (Memorial Sloan Kettering Cancer Center), and UCSC Genome browser have been developed to help bridge the gap between genomic studies and clinical results.

Digital Expression Explorer 2 (DEE2) was launched in 2019 by Deakin University and the Walter and Eliza Hall Institute of Medical Research. This web-based, free-of-charge, open repository of RNA-seq data in the form of gene-level and transcript-level expression counts, contains a large volume of uniformly processed RNA-seq data from various organisms. This platform offers several advantages over its predecessor, DEE1. Specifically, DEE2 contains over 5.3 trillion assigned reads from 580,000 RNA-seq datasets, covering nine different species. All data in DEE2 undergoes standardized processing steps, ensuring consistency and facilitating comparative analysis across datasets. 

## 6. Microarray/RNAseq Data Repositories

Sequencing technologies have improved the analysis and understanding of genomic research studies, especially in targeted therapy development and oncologic biomarker identification. DNA microarray and RNAseq technology allow for the stratification of patients, prognosis prediction, and improvement of early diagnostic methods using bioinformatics. Both technologies can be used to create publicly available data on repositories such as Gene Expression Omnibus (GEO). GEO is a public repository comprised of over 4000 high throughput gene expression, microarray, and hybridization array data sets available to researchers. Similar repositories exist globally, such as the European Array Express. Array Express has acquired over 44TB of data. The data on these repositories can be downloaded using R Bioconductor packages.

Bioconductor is an open-source software that analyzes genomic data generated by molecular experiments. This software is primarily based on the R programming language and is used for high-throughput data, integration, statistical analysis, data pre-processing, and visualization.

### Integration of Multiple Repositories for Cancer Research

Highlighting two of the previously mentioned repositories, TCGA and GEO, both of which are widely used in cancer research. GEO is a public functional genomic repository comprised of multiple data types, including gene expression, genomic hybridization, and sequencing across different experimental conditions and organisms. GEO houses a large amount of transcriptomic data and is freely accessible. This facilitates its widespread use by researchers. In contrast, TCGA has a specialized focus on cancer, providing detailed multi-omics data specific to cancer types. By leveraging both GEO’s extensive transcriptomic data and TCGA’s comprehensive cancer-specific datasets, researchers gain a more holistic view of the genomic landscape and provide the framework for clinically applicable results. Using both repositories enables a deeper understanding of gene expression patterns and genomic alterations in cancer, ultimately aiding in the development of targeted therapies and personalized medicine. For example, A 2022 study on skin cutaneous Melanoma (SKCM) integrated single-cell RNA sequencing data from GEO with bulk RNA sequencing data from TCGA. This combined approach enabled the identification of long-noncoding RNA (lncRNA) PRRT3-AS1 as a significant biomarker for SKCM [42].

## 7. Data Manipulation and Structuring

Data manipulation and structuring are essential processes in data analysis and management. Raw data often contains missing values, errors, or outliers. Data manipulation allows for early-stage quality control and pre-processing of the data, making it suitable for analysis. Data may come from various sources that utilize different protocols and formats. For analysis, the data needs to be converted into a consistent format. This can help improve the reproducibility of the experiment. Lastly, feature engineering can also be a part of data manipulation by generating new variables (features) from existing variables. This allows researchers to capture important patterns and relationships in the data that may not be initially apparent. Tools such as SAMtools, Picard, BEDtools, Pandas, dplyr, SQL, Apache Spark, awk, and SED are used in genomic data manipulation.

Data may also undergo transformation. Transformation involves converting data formats from one to another. For example, Fastq files may be converted to SAM/BAM files or VCF (Variant Call Format). In addition, large data may be summarized or aggregated into more manageable forms. In time-series data, this may allow for an observation of trends that may not have been revealed in high-frequency datasets. Similarly, data can be merged/joined by common identifiers, allowing for enriched analysis. Merging uncovers relationships between data variables in different tables or matrices.

### Structuring

Normalizing the data is also important for the experimental design. This involves scaling data to a standard range to help compare variables with different scales, such as RPKM/FPKM or TPM in RNA-Seq studies. It reduces the impact of outliers that may alter the results. Likewise, logarithmic transformation proves invaluable when tackling data with skewed distributions, enabling more effective application of certain statistical analyses. 

There are several methods to consider for structuring genomic data. Variant encoding, for instance, entails standardizing genetic variants and can be achieved through commonly employed tools like HGVS notation, VCFtools, Bcftools, and PLINK. Genomic interval structuring, on the other hand, organizes data on the basis of genomic coordinates, and this can be efficiently carried out using BEDtools, GATK, VEP, and UCSC Genome Browser. Furthermore, data can be enriched with annotations such as gene names, associated phenotypes, and other functional impacts, which can be accomplished using tools like ANNOVAR, SnpEff, VEP, and BioMart.

## 8. Data Analysis

Bioinformatic tools are used to analyze and visualize the large datasets derived from the data repositories. According to researchers, cBioPortal is one of the most common tools to analyze cancer genomic studies. This multi-omics analysis tool contains pre-processed data from over 147 cancer studies that permit the interpretation of somatic mutations, race/ethnicity, gene expression, copy number changes, methylation, and phosphorylation. This tool also has heatmap and gene network visualization capabilities. Other web-based or desktop applications that allow visualization include the UCSC Genome Browser, Integrative Genomics Browser (IGV), and the Catalogue of Somatic Mutations in Cancer (COSMIC). Notably, COSMIC is one of the most comprehensive databases for investigating known somatic mutations in cancer. The Gene Ontology Consortium (GOC) aims to develop computational models across biological systems [43,44]. Gene Ontology (GO) provides both human-readable and machine-readable information about the functions of genes [43,44]. Other tools for analyzing somatic mutations and their functional impact include SIFT, PolyPhen, Mutation Assessor, and IntoGen [45,46,47].

### Pathway and Network Analysis Tools

Pathway analysis incorporates a variety of applications and programs to analyze and interpret biological data such as genes and proteins. This allows researchers to critically analyze interactions between genes, proteins, metabolites, and other biological regulatory molecules. It is important to note the difference between pathway analysis and network analysis. Pathway analysis is often a visual conceptualization of widely studied research [48]. Network analysis includes genome and proteome-wide interactions [48].

Qiagen Ingenuity Pathway Analysis (QIAGEN IPA) is a commercial tool that enables the interpretation of omics data by identifying relevant pathways, networks, and functional annotations [49]. It provides visualization and analysis of molecular interactions. Gene Set Enrichment Analysis (GSEA) assesses whether a predefined set of genes shows statistically significant differences between different experimental conditions [50]. It is widely used to identify enriched pathways using gene expression data [50]. In addition, Reactome is a free and open-source pathway database that provides curated and peer-reviewed pathway analysis and visualization resources [51,52].

Furthermore, the Kyoto Encyclopedia of Genes and Genomes (KEGG) is a comprehensive resource for biological pathways and functional annotations [53]. It includes a pathway mapping tool that helps visualize omics data in the context of pathways [53]. DAVID (Database for Annotation, Visualization, and Integrated Discovery) provides functional annotation tools to understand the biological significance of large gene lists [54]. It offers pathway analysis, gene ontology enrichment, and functional annotation clustering [54]. In addition, the Pathway Studio 9.0 software allows the integration of molecular and clinical data for pathway analysis [55]. It aids in building and visualizing pathway models and supports network analysis. Metacore is a web-based platform focusing on pathway analysis, network building, and visualization [56]. It provides tools for omics data interpretation and helps uncover hidden relationships in complex biological systems. STRING is a database and web resource that analyzes protein-protein interactions and provides functional enrichment analysis [57]. It can be used to generate interaction networks and identify enriched pathways. R-based Bioconductor packages such as Pathview, ClusterProfiler, and fgsea provide various tools for pathway analysis, gene set enrichment, and visualization [58,59,60]. Although mainly used for network visualization, Cytoscape also supports pathway analysis plugins that allow users to analyze and visualize pathways and networks simultaneously [61].

Similarly, WebGestalt is an online tool that supports functional enrichment analysis, pathway analysis, and network visualization of omics data [62,63]. Lastly, PantherDB is a resource that offers tools for gene ontology analysis, functional annotation, and pathway analysis. It covers a wide range of species and biological functions. In addition to the many pathway analysis tools, Table 1 provides a brief overview of some gene network analysis tools. There are vast possibilities with the features of these tools in conjunction with different types of omics data.

## 9. Using Predictive Algorithms

**Figure 3:**
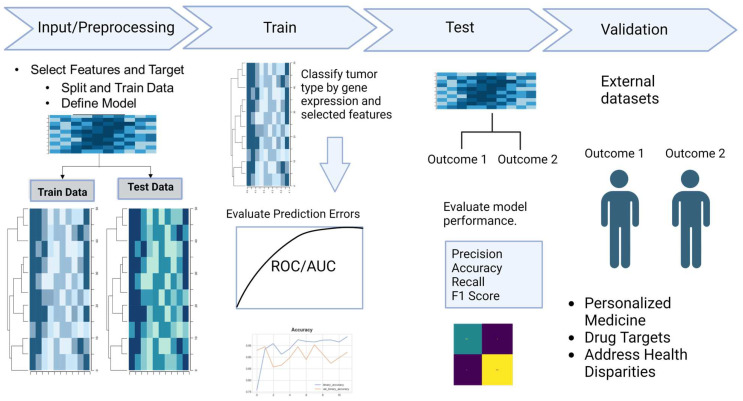
Overview of a classification algorithm workflow. Classification algorithms utilize features to identify patterns in the input data. During the training stage, the model uses statistical calculations to develop predictions. In the testing stage, the initial model is employed to evaluate its performance using precision, accuracy, recall, and F1 score. The final step is to validate the model on external datasets and address various scientific questions.

The identification of predictive biomarkers plays a pivotal role in advancing our understanding of complex diseases like cancer. ML techniques have emerged as valuable tools for this purpose. In Figure 3, we delve into the multifaceted process of discovering predictive biomarkers in cancer research using ML algorithms.

### 9.1. Data Labeling and Supervised Learning

To uncover predictive biomarkers, researchers employ supervised learning methods in ML. Supervised learning algorithms utilize labels to learn data patterns to generate a labeled output. In this context, the labels represent meaningful associations with specific values. For instance, in a cancer study, these labels could signify critical information such as tumor status (positive or negative), patient outcomes (survival or recurrence), or molecular subtypes. These labels are often encoded using a binary system of 0 and 1 to provide a negative or positive context for a data value.

### 9.2. Data Labeling Beyond Cancer Research

The concept of data labeling extends beyond cancer research. In various domains like computer vision, natural language processing, and speech recognition, labels are used to categorize and make sense of data. For example, in computer vision, labels might represent object classes in an image, while in natural language processing, they can indicate sentiment (positive or negative) in text analysis.

### 9.3. Data for Predictive Models

For cancer research studies, the input values for predictive models typically consist of matrices representing untreated biological conditions, such as gene expression profiles. These datasets are often characterized by high dimensionality due to the many genes and features.

### 9.4. Training ML Models

ML models leverage statistical computations to train classification algorithms on input and labeled data. When applied to cancer research, these models can help identify significant genes, biomarkers, or patient subgroups associated with disease progression, prognosis, or response to therapy. Notably, a wide range of ML and deep learning (DL) algorithms can be employed depending on the research question and the nature of the data.

### 9.5. Machine-Learning Tools and Languages

Python is commonly used for many ML and DL developments. However, R packages, such as Caret for pre-processing and Modelr for modeling, can also be used effectively for algorithm development. TensorFlow v2.17.0 is an open-source software library for numerical computation and ML. It offers various pre-built models and tools for RNA-seq analysis [64]. PyTorch is another open-source ML framework popular for its flexibility and ease of use [65]. It also provides numerous resources for RNA-seq analysis. DeepSEA2 is a deep learning model for predicting regulatory elements in DNA sequences [66]. Similarly, DeepSEA++ is an advanced model of DeepSEA2 that can predict regulatory elements across multiple cell types. SpliceAI is a deep learning (DL) model for predicting alternative splicing events in RNA transcripts [67]. It analyzes RNA sequences and uses a deep convolutional neural network to identify potential splice sites. SpliceAI can predict both known and unknown splicing events with high accuracy [67]. rna2vec is a tool that uses word2vec, a natural language processing technique, to embed RNA sequences into vector representations [68]. These vectors capture the underlying biological meaning of the RNA sequences and allow for efficient downstream analysis tasks. Examples include clustering RNA sequences based on their similarity and functional categories and identifying regulatory elements in RNA sequences; DeepRibo is another DL model for predicting RNA-binding protein targets [69].

### 9.6. Validation and Reproducibility of ML Models

ML has emerged as a powerful tool in cancer research, offering significant potential for improvements in multi-modal data analysis. However, the effectiveness of these models relies heavily on validation and reproducibility, two crucial aspects that ensure their reliability and generalizability. Researchers must consider validation methods throughout the predictive model development process to prevent overfitting or underfitting the data. Ensuring reproducibility is equally critical, as it enables the scientific community to verify and build upon research findings.

Predictive algorithms are indispensable tools in cancer research, aiding in identifying crucial biomarkers and insights into the disease. By leveraging ML techniques, researchers can unlock valuable knowledge contributing to better diagnostics, treatment strategies, and improved patient outcomes. Validation can prevent overfitting, which occurs when a model learns the training data too well, failing to generalize to unseen data. In contrast, underfitting happens when a given model is too simple to capture the underlying relationships in the data. Appropriate validation also ensures model accuracy and generalizability. This assesses model performance in real-world scenarios and provides insights into its generalizability to different populations and contexts. Common ML model validation methods include the Hold-Out Method, which divides data into training and testing sets; K-fold Cross-Validation, which repeatedly splits data into training and testing folds for better evaluation; and Leave-One-Out Cross-Validation, which uses each data point as a test case [70,71,72].

Reproducibility enables verification of analysis results. Indeed, reproducible research allows others to replicate a study, verify findings, and build upon them. There are several challenges to reproducible ML models. There is a lack of standardized data analysis ML pipelines. Different researchers often use different tools and parameters, leading to inconsistencies. Limited data-sharing practices due to data access restrictions hinder reproducibility and collaboration. Researchers and developers must clearly document their methods, data sources, and code to facilitate reproducibility. Predictive algorithms are indispensable tools in cancer research, aiding in identifying crucial biomarkers and insights into the disease [73]. By leveraging machine learning techniques, researchers can unlock valuable knowledge contributing to better diagnostics, treatment strategies, and improved patient outcomes.

## 10. Applications of Bioinformatics Tools in Cancer Research

As shown in Table 2, bioinformatics tools are pivotal in advancing cancer research by providing in-depth analyses and comprehensive perspectives on the complex molecular mechanisms underlying cancer development, progression, and treatment. These tools enable the integration and interpretation of large volumes of multi-omics datasets and clinical data, allowing researchers to identify key genetic alterations, oncogenic pathways, and potential therapeutic targets. In cancer genomics, bioinformatics tools aid in identifying somatic mutations, copy number variations, and driver mutations that contribute to tumorigenesis. Additionally, transcriptomics analysis helps uncover dysregulated gene expression patterns associated with specific cancer types, stages, and ethnic/racial groups. Through network analysis, researchers can elucidate intricate gene-gene interactions and signaling pathways involved in the underlying molecular processes.

Moreover, bioinformatics can facilitate the prediction of drug responses, survival analysis, and the discovery of personalized treatment options by investigating patient-specific genetic profiles. Bioinformatics tools bridge the gap between basic research and its clinical applications, providing clinicians with valuable insights for early diagnostics, prognosis, and therapeutic options. Ultimately, this knowledge contributes to the advancement of precision oncology and the improvement of patient outcomes.

## 11. Ethics in Bioinformatics

The integration of bioinformatics into biomedical research and clinical practice has led to unprecedented advancements in understanding and treating complex diseases like cancer. However, these advancements also bring forth significant ethical considerations. Ethical considerations in bioinformatics encompass issues related to data privacy, informed consent, data sharing, the potential misuse of genetic information, and ethics in AI and machine learning. Addressing these ethical challenges is crucial to ensuring the responsible use of bioinformatics in research and healthcare.

### 11.1. Data Privacy and Security

A primary ethical concern in bioinformatics is the privacy and security of patient data [74]. High-throughput technologies and bioinformatics tools generate large amounts of genomic and clinical data, which if not protected, can lead to confidentiality breaches. To protect patient identity, data must be anonymized or de-identified before being shared or published [75,76]. However, genomic data, due to its unique nature, poses challenges in achieving true anonymization [77]. Advanced techniques and stringent protocols are required to ensure individual identities cannot be re-identified from genomic data. Another avenue to ensure data privacy is data encryption [78,79]. Encryption technologies and careful data management can be used to safeguard data against unauthorized access during storage and transmission [79,80]. This includes using secure servers and encrypted communication channels. Additionally, implementing access control mechanisms may provide a physical safeguard only permitting authorized personnel access to sensitive data.

### 11.2. Informed Consent

Informed consent is an ethical consideration in biomedical research. Participants must be fully informed about the nature of the research, the type of data collected, how the data will be used, and potential risks and benefits. In the context of bioinformatics, informed consent involves navigating the complexity of information, broad consent, and reconsent. Explaining complex genomic research and bioinformatics analysis to research participants can be challenging. Researchers must ensure that consent forms are clear and comprehensive and provide sufficient detail about the study’s aims and methodologies. Researchers may opt for broad consent where participants may agree to allow researchers to use their data for future studies. Although this may be beneficial for scientific research, participants’ autonomy and their right to withdraw consent must be acknowledged. Similarly, if the scope of the research or data use changes, re-consent may be necessary to respect the participants’ autonomy.

### 11.3. Data Sharing

Data sharing is a key component of bioinformatics. However, establishing clear data sharing agreements is essential for protecting participants’ rights and responsible use of the data. Many data repositories like dbGap (Database of Genotypes and Phenotypes) offer both open access and controlled access data [81]. Open access data promotes transparency while controlled access allows researchers to access the data while maintaining participant confidentiality.

### 11.4. Potential Misuse of Genetic Information

Utilizing genomic data for research poses significant ethical risks, including genetic discrimination and stigmatization. It is important to consider that individuals might be subjected to discrimination based on their genetic information. In the United States, the Genetic Information Nondiscrimination Act (GINA), aims to protect individuals, but continuous vigilance is required to enforce and update these protections [82]. Furthermore, certain genetic findings can lead to the stigmatization of individuals or groups. Especially if associated with specific diseases or conditions. Additionally, researchers must be aware of the health disparities, ensuring that genomic research does not disproportionately impact or overlook vulnerable populations and that findings are applied equitably.

### 11.5. AI and Machine Learning

Prediction models and artificial intelligence in bioinformatics can increase the analytical capacity by being able to manage significantly large datasets. However, this technology brings ethical considerations. AI models can inherit biases present in training data, leading to biased outcomes [83]. Using diverse datasets and implementing fairness-aware algorithms (FAIs) is crucial to mitigate bias and ensure equitable treatment of all population groups [83,84,85,86,87].

The complexity of AI models often leads to a lack of transparency. Assuring transparency in AI decision-making processes and maintaining accountability for AI-generated results are essential for maintaining trust in bioinformatics applications [87]. 

Ethical considerations in bioinformatics are multifaceted and require ongoing attention as the field evolves. Ensuring data privacy and security, obtaining informed consent, responsible data sharing, preventing the misuse of genetic information, and addressing the ethical challenges in AI are all crucial components of ethical bioinformatics practice.

## 12. Advancements, Challenges, and Future Directions

The rapid advancements in high-throughput technologies, specifically next-generation sequencing, have transformed cancer research. The use of bioinformatics tools has paved the way for significant discoveries in genomic biomarkers and precision oncology. These technologies highlight the great potential for advancing personalized therapies, improving diagnostic methods, and addressing health disparities. However, there are several challenges and future directions to be considered.

### 12.1. Key Findings and Applications

Bioinformatics tools have been instrumental in identifying and validating biomarkers in cancer genomics. Platforms like TCGA, GDC, and ICGC provide vast repositories of multi-omics data facilitating comprehensive analyses of genetic alterations, gene expression, and epigenetic modifications. Tools like cBioPortal and COSMIC have enabled the exploration of somatic mutations and their implications in tumorigenesis. Furthermore, RNA-Seq workflows, including tools such as FastQC and HISAT2, have advanced our understanding of transcriptomic challenges in cancer studies.

The application of machine learning in predictive biomarker discovery has grown significantly in recent years. By leveraging large datasets, ML algorithms can unveil complex patterns that contribute to the prediction of disease outcomes and treatment responses. Tools like TensorFlow and PyTorch have been used to develop models for RNA-Seq analysis, contributing to the identification of significant biomarkers and patient subgroups. For example, a study used TensorFlow to build a deep learning model for classifying cancer types on the basis of RNA-Seq data. The model demonstrated high accuracy in distinguishing between various cancer types [88].

### 12.2. Data Challenges

Biomarker discovery often involves the integration of various types of omics data, such as genomics, transcriptomics, proteomics, and metabolomics. Therefore, ensuring the quality, accuracy, and compatibility of these heterogeneous datasets can pose a challenge. Cancer datasets frequently exhibit issues such as incomplete data, batch effects, noise, and variability due to differences in sample collection, processing, and sequencing technologies. These inconsistencies can lead to unreliable results and affect the accuracy of the downstream analyses. In addition, using large high-dimensional omics data may lead to overfitting or false discoveries. Multiple hypothesis testing and data mining can increase the likelihood of identifying spurious correlations that fail validation. Normalization, batch correction, and rigorous statistical methods are essential to reliable and reproducible findings.

### 12.3. Challenges in Biomarker Identification, Validation, Clinical Implications, and Ethics

Biological systems are complex, and bioinformatics used for biomarker discovery must account for multi-omics interactions between genes, proteins, and other molecules in pathways and networks. The intricate nature of bioinformatics workflows, often involving multiple software and specific parameter settings, is another challenge for reproducibility. Ensuring reproducibility requires meticulous documentation and adherence to standardized protocols.

As biomarkers are identified, they must be validated in independent cohorts or experimental settings to ensure their reproducibility and clinical relevance. However, many initial biomarker candidates fail to reproduce the same results in validation experiments, emphasizing the need for rigorous validation processes. Transitioning from biomarker discovery to clinical implementation requires validation, regulatory approval, and demonstration in a clinical setting. Clinical trials and regulatory considerations can add significant time and cost. In bioinformatics studies, handling patient data and information raises ethical and privacy concerns. Researchers must implement data security protocols, informed consent, and compliance with privacy regulations in their studies.

### 12.4. Health Equity in Multi-Modal Cancer Research Challenges

While multi-modal data analysis has the potential to revolutionize cancer research and improve patient outcomes, several challenges hinder its application in a way that promotes health equity. Underserved communities often lack access to quality healthcare, technology, and precision medicine (e.g., CLIA NGS) resulting in limited data collection and representation in multi-modal datasets. This can lead to biased models that are inaccurate for diverse populations. Historical biases in healthcare and data collection can perpetuate inaccuracies and misrepresentations of certain groups, which can lead to discriminatory outcomes and widen health disparities. 

ML algorithms can inherit and amplify biases present in training data, leading to discriminatory predictions (i.e., algorithm bias) and unfair outcomes for certain groups. Understanding how algorithms make decisions and the reasons behind their predictions is crucial for identifying and mitigating biases. However, many artificial intelligence (AI)/ML models lack transparency and interpretability, making it difficult to assess and address bias. Ensuring ethical and fair use of multi-modal data requires robust regulatory frameworks and oversight. Unfortunately, current regulations or practices lag behind technological advancements. 

### 12.5. Future Directions and Emerging Technologies

The future of bioinformatics in precision oncology is set to leverage emerging technologies and methodologies to overcome current limitations. As cancer research continues to generate large complex datasets, integrating multi-omics approaches, advanced machine learning algorithms, and innovative sequencing technologies will be essential. These advancements will not only advance our understanding of cancer biology, but also refine biomarker discovery, improve predictive models, and ultimately enable more personalized and effective treatments for patients. These future directions will help bridge the gap between research and clinical practice, leading to new discoveries and improved patient outcomes.

#### 12.5.1. Single-Cell and Spatial Omics

Single-cell RNA sequencing (scRNA-seq) and spatial transcriptomics are improving our understanding of cellular heterogeneity. These technologies allow for high-resolution analysis of gene expression at the individual cell level, investigating the diversity of cell types and interactions within the microenvironment [89]. However, they present bioinformatic challenges including data integration, scalability, and standardization. Advanced computational methods are required to combine scRNA-seq and spatial transcriptomics data while retaining both high-resolution gene expression information and spatial context. The high-dimensional nature of the data requires robust bioinformatics tools for meaningful analysis. Despite these challenges, these technologies offer opportunities for advancement and innovation. They provide detailed molecular profiling that supports personalized medicine by tailoring treatments according to individual disease characteristics. 

#### 12.5.2. Long-Read Sequencing Technologies

Long-read sequencing technologies, such as PacBio and Oxford Nanopore, offer the capability to sequence full-length transcripts, providing more accurate identification of isoforms and gene fusions in single-cell and spatial transcriptomics data. This technology is beneficial for identifying novel isoforms, gene fusions, and complex structural variations that may be overlooked by short-read technologies. Although long-read sequencing technologies have their advantages, they may face challenges related to higher error rates compared to short-read sequencing technologies. Bioinformatics tools must be adapted to address the errors and efficiently process the data. Additionally, the computational demands for analyzing long-read data are substantial, necessitating advanced algorithms and computing infrastructure.

#### 12.5.3. Machine Learning Approaches

Machine learning is increasingly being applied to analyze complex multi-omics, single-cell, and spatial omics datasets. These methods enable the identification of rare cell populations, cell-cell, interactions, and spatial patterns of gene expression. Machine learning and artificial intelligence can be used to uncover patterns and clinical outcome predictions on the basis of multi-omics profiles.

#### 12.5.4. Clinical Integration

Combining single-cell and spatial omics data with clinical information can help identify biomarkers, predict treatment responses, and develop personalized therapies for cancer patients. By correlating molecular data with clinical outcomes, researchers generate actionable insights to inform treatment strategies and improve patient care.

The integration of emerging technologies in precision oncology requires the continuous development of bioinformatics tools and frameworks. Key areas for future research and development include multi-omics integration, enhanced visualizations, machine learning, and AI. Multi-omics integration involves developing sophisticated methods for integrating multi-omics data to provide a holistic view of cellular function and interactions. Visualization tools may help aid the interpretation of complex data findings. Addressing these challenges and leveraging the opportunities presented by emerging technologies will enhance our understanding of cancer biology and make strides toward understanding the potential of biomarkers in personalized healthcare.

## Figures and Tables

**Table 1:** Gene network analysis tools used in bioinformatics research studies.

Network Analysis Tool	Description
*ARCANE*	A tool for inferring gene regulatory networks from gene expression data. It employs mutual information-based methods to identify direct regulatory relationships.
*WGCNA* (Weighted Gene Co-Expression Network Analysis)	Used to identify co-expression modules within large gene expression datasets. It helps uncover gene networks related to specific biological processes or conditions.
*GeneMANIA*	A tool integrates various data sources to predict and visualize gene function and interactions. It helps users understand the functional relationships between genes in the context of specific biological processes.
VisANT	Network visualization and analysis tool that enables the exploration of biological pathways, gene interactions, and molecular networks
BioGRID	Offers tools for network analysis. It helps users explore physical and genetic interactions within a network context.
NetworkAnalyst	Integrated platform for network-based analysis that supports various types of omics data. It provides tools for network visualization, enrichment analysis, and pathway analysis.
RegulatoryNetworks	Focuses on the reconstruction and analysis of gene regulatory networks. It utilizes transcription factor binding site data to infer regulatory interactions.
GRNsight	Web-based tool for visualizing and analyzing gene regulatory networks. It helps users explore transcriptional interactions and regulatory relationships.
CytoScape.js	JavaScript library for network visualization that can be integrated into web applications to display and analyze gene networks interactively.
PathVisio	Offers plugins for network analysis. It allows users to draw, edit, and analyze biological pathways and networks.

**Table 2:** Overview of bioinformatics tools used in cancer research genomics.

		URL	Description
**Repositories**	TCGA	https://www.cancer.gov/ccg/research/genome-sequencing/tcga (Accessed on 31 October 2023)	Multi-omics data of 20,000 patients and 33 tumor types
	ICGC	https://dcc.icgc.org/ (Accessed on 31 October 2023)	55 cancer genomics projects with tools to analyze and visualize data.
	GDC	https://gdc.cancer.gov/ (Accessed on 31 October 2023)	Developed by the NIH and NCI and includes TCGA AND TARGET
	Gene Expression Omnibus (GEO)	https://www.ncbi.nlm.nih.gov/geo/ (Accessed on 31 October 2023)	Stores processed data files, including RNA-Seq and chip-Seq
	Array Express	https://www.ebi.ac.uk/biostudies/arrayexpress (Accessed on 31 October 2023)	Stores high-throughput genomics data.
	European Nucleotide Archive (ENA)	https://www.ebi.ac.uk/ena/browser/home (Accessed on 31 October 2023)	Stores raw data files in Fastq format.
	Sequence Read Archive	https://www.ncbi.nlm.nih.gov/sra (Accessed on 31 October 2023)	Stores raw data files in SRA format.
	Dryad Digital Repository	https://datadryad.org/stash (Accessed on 31 October 2023)	Open access repository of medical research data
	Figshare	https://figshare.com/ (Accessed on 31 October 2023)	Cross-disciplinary open-access repository for academic research
	Harvard Dataverse Network	https://dataverse.harvard.edu/ (Accessed on 31 October 2023)	Multi-disciplinary data storage center
	Kaggle	https://www.kaggle.com/ (Accessed on 31 October 2023)	Platform for data science training, competitions, and datasets
	Network Data Exchange	https://home.ndexbio.org/about-ndex/ (Accessed on 31 October 2023)	Repository for network biology data
	Open Science Framework	https://osf.io/ (Accessed on 31 October 2023)	Platform for collaborating on research projects
	GenoVault	https://github.com/bioinformatics-cdac/GenoVault (Accessed on 31 October 2023)	Cloud-based repository for NGS data
	UK Biobank	https://www.ukbiobank.ac.uk/ (Accessed on 31 October 2023)	Large-scale biomedical research database
**Tools for data analysis and visualization**	cBioPortal	https://www.cBioPortal.org/ (Accessed on 31 October 2023)	Visualizations, analysis, cancer genomics projects
	COSMIC	https://cancer.sanger.ac.uk/cosmic (Accessed on 31 October 2023)	Database of somatic mutations in cancer.
	IGV	https://software.broadinstitute.org/software/igv/ (Accessed on 31 October 2023)	High-performance genome browser for visualizing and analyzing large-scale genomic data.
	Regulome Explorer	https://explorer-cancerregulome.systemsbiology.net/ (Accessed on 31 July 2024)	Exploring and analyzing regulatory elements in the genome.
	UCSC Genome Browser	https://genome.ucsc.edu/ (Accessed on 31 October 2023)	Provides access to a vast collection of genomic data and annotations
	Bioconductor	https://www.bioconductor.org/ (Accessed on 31 October 2023)	Open-source software project for the analysis and comprehension of high-throughput genomics data.
	Cytoscape	https://cytoscape.org/ (Accessed on 31 October 2023)	Network analysis and visualization tool
	Gene Ontology	http://geneontology.org/ (Accessed on 31 October 2023)	Standardized system for annotating genes and their functions in different organisms.
	UALCAN	https://ualcan.path.uab.edu/ (Accessed on 31 October 2023)	Web portal for in-depth analysis of cancer transcriptome data.
	DAVID	https://david.ncifcrf.gov/ (Accessed on 31 October 2023)	Functional annotation and enrichment analysis of gene lists
	HumanBase (GIANT)	https://hb.flatironinstitute.org/ (Accessed on 31 October 2023)	Exploring human genomic data and conducting large-scale integrative analysis.
	CEDER	https://ieeexplore.ieee.org/document/6205734 (Accessed on 31 October 2023)	Detection of differentially expressed genes
	CPTRA	https://pubmed.ncbi.nlm.nih.gov/19811681/ (Accessed on 31 October 2023)	Package for analyzing transcriptome sequencing data
	Bioconductor	https://www.bioconductor.org/ (Accessed on 31 October 2023)	Open-source software for genomic data analysis
**Tools for analyzing biomarker signatures from omics data**	Limma	https://bioconductor.org/packages/release/bioc/html/limma.html (Accessed on 31 October 2023)	Statistical package for the analysis of microarray and RNA-seq data.
	Caret	https://cran.r-project.org/web/packages/caret/index.html (Accessed on 31 October 2023)	R package for training and evaluating ML models.
	netClass	https://doi.org/10.1093/bioinformatics/btu025 (Accessed on 31 October 2023)	A tool for classifying biological samples using network-based features.
	WGCNA	https://horvath.genetics.ucla.edu/html/CoexpressionNetwork/Rpackages/WGCNA/	Identifying gene modules and their relationships in high-throughput data.
**Somatic variants interpretation**	MyCancerGenome	https://www.mycancergenome.org/ (Accessed on 31 October 2023)	Understanding cancer genomics and personalized cancer treatment options.
	Civic	https://civicdb.org/welcome (Accessed on 31 October 2023)	Treatment options for cancer patients based on their unique tumor DNA
	TARGET	https://www.cancer.gov/ccg/research/genome-sequencing/target (Accessed on 31 October 2023)	Molecular characterization
	CGI	https://www.genomicinterpretation.org/ (Accessed on 31 October 2023)	Genomic alterations in cancer and their potential clinical relevance
	ClinicalTrials.gov	https://www.clinicaltrials.gov/ (Accessed on 31 October 2023)	An online database that provides information on clinical trials
	EUCTR	https://www.clinicaltrialsregister.eu/ (Accessed on 31 October 2023)	Database containing information on clinical trials conducted in the European Union

## Data Availability

No new data was created in this literature review.
